# Prevalence of work related musculoskeletal disorders (WMSDs) and ergonomic risk assessment among readymade garment workers of Bangladesh: A cross sectional study

**DOI:** 10.1371/journal.pone.0200122

**Published:** 2018-07-06

**Authors:** Mohammad Didar Hossain, Afzal Aftab, Mahmudul Hassan Al Imam, Ilias Mahmud, Imran Ahmed Chowdhury, Razin Iqbal Kabir, Malabika Sarker

**Affiliations:** 1 James P. Grant School of Public Health, BRAC University, Mohakhali, Dhaka, Bangladesh; 2 Foundation for Advancement of Innovations in Technology and Health (faith), Dhaka, Bangladesh; 3 Centre for the Rehabilitation of the Paralysed (CRP), CRP-Chapain, Savar, Dhaka, Bangladesh; 4 College of Public Health and Health Informatics, Qassim University, Bukayriah, Qassim, The Kingdom of Saudi Arabia; 5 Institute of Public Health, University of Heidelberg, Heidelberg, Germany; West Virginia University, UNITED STATES

## Abstract

**Background:**

Work related Musculoskeletal Disorders (WMSDs) are one of the most common occupational diseases which mainly affects the lower back, neck and upper and lower extremities. The aim of this study was to determine prevalence of WMSDs in nine body regions among Ready Made Garment (RMG) workers in Bangladesh and ergonomics assessment of their exposure to risk factors for the development of WMSDs.

**Methods:**

This cross-sectional study was conducted among 232 RMG employees (male: 46; female: 186; age: >18yrs) from nine RMG factories in Dhaka division during October 2015 to February 2016. Data were collected using a structured questionnaire consist of demographic questions, Nordic Musculoskeletal Questionnaire-Extended (NMQ-E) for WMSDs assessment in nine body regions and Quick Exposure Check (QEC) method for ergonomic assessment. Prevalence of WMSDs for each body region was determined. The association between WMSDs and ergonomic assessment of their exposure to risk factors were also analyzed.

**Results:**

Respondents’ mean age was 31.3 years (SD = 7). Their mean Body Mass Index (BMI) was 23.51 kg/m^2^ (SD = 3.74). Among 186 female respondents, 46 reported lower back pain (24.7%) and 44 reported neck pain (23.7%). Among 46 male respondents, 10 reported neck pain (21.7%) while 6 reported knee pain (13%). Statistically significant relationship was found between twelve month WMSDs in anatomical region in elbows (p = 0.02), hips (p = 0.01), knees (p = 0.01) and ankle (p = 0.05) with age; upper back (p = 0.001), elbows (p = 0.001), wrists (p = 0.03), hips (p = 0.001) and ankles (p = 0.01) with job experience; hips with BMI (p = 0.03); elbows (p = 0.04) with daily working hour. QEC assessment showed that level of exposure to WMSDs risk was high among 80% of the study population (p<0.003).

**Conclusion:**

The study found that lower back and neck were the most affected areas among RMG workers. Moreover, QEC findings warned the level of exposure to WMSDs risks is high and ergonomics intervention along with investigation and change to decrease exposure level is essential. Addressing musculoskeletal risk factors through ergonomic interventions in terms of working space, workers sitting/standing posture, seat and hand position during work and work-rest cycle are encouraged in RMG sector and policy makers.

## Background

Low back pain (LBP), neck pain and other Musculoskeletal Disorders (MSDs) are the leading causes of years lived with disability (YLDs) [1]. LBP ranked highest in terms of disability (YLDs), and sixth in terms of Disability-adjusted life-years (DALYs) in the Global Burden of Disease 2010 Study [2]. Several epidemiological studies have demonstrated evidence of a causal relationship between physical exertion at work and Work related Musculoskeletal Disorders (WMSDs) [3]. Common risk factors of WMSDs are awkward postures, prolonged static work, repetitive movements, manual material handling, forceful exertions and vibration [4–6]. In addition, job dissatisfaction, stress at work and time pressure comprise major psychosocial factors related to WMSDs [7, 8]. WMSDs not only substantially deteriorate physical and emotional health and productivity of the industrial work force [5, 9, 10] but also the most expensive form of work disability [11–13].

Readymade Garment (RMG) industries are the main source of earning foreign currencies in Bangladesh. RMG exports US$24.5 billion (2013–14) accounting for over 80% of the nation’s export earnings [14]. In 2011–12, there were 5,400 RMG factories in Bangladesh which employed 4 million workers [15, 16]. In 2015–16, number of factories reduced but still 4 million workers were employed [16] of which 55–60% are women [14].

There is a dearth of literature regarding WMSDs including ergonomic assessment of RMG worker’s exposure to risk factors for the development of WMSDs. Only a limited number of studies from Bangladesh [17–20] reported musculoskeletal disorders and work related ergonomic risk factors despite heterogeneous tools used. The aim of this study was to measure prevalence of WMSDs in nine body regions (neck, shoulders, upper back, elbows, wrist/hand, lower back, hips/thighs, knees and ankles/feet) among RMG workers in Bangladesh and ergonomic assessment of their exposure to risk factors for the development of WMSDs.

## Materials and methods

### Study design, study site and setting

This cross sectional study was a part of larger mixed method study using exploratory embedded design conducted three areas in Dhaka division- Sreepur, Narayanganj and Mirpur. According to the Government of Bangladesh database, there are a total of 4,809 RMG factories in eight divisions of Bangladesh [21] of which 1,961 are in Dhaka division. This division was chosen for having the highest geographical concentration of RMG factories across Bangladesh; covering more than 40% of the total RMG factories and a higher concentration of workers engaged in garment industry than other divisions.

A total number of 28 RMG factories were randomly selected. A formal ‘letter of invitation’ was provided to the authority of the preliminary selected 28 RMG factories who agreed to participate in this study. After comprehensive explanation of study objectives and study plans, among the 28 factories, nine provided permission to recruit their workers. A list of both male and female workers and a formal ‘letter of support’ was collected from the respective RMG factories.

### Study period

The study was conducted from October 2015 to February 2016.

### Study population

Study population comprised of male and female who had at least 10 years of working experience in RMG sector.

### Sample size calculation

Sample size was calculated according to WHO Guideline [22]. To determine the representative sample size, the formula: n = Z^2^P(1-P)/d^2^ was used (page 2). With 5% precision level, and 95% confidence interval (CI), our sample size was 232 RMG workers. Literature review till 2015 revealed that approximately 80% of the workers in the RMG sector are female. Study population of 45,211 was obtained from the selected nine RMG factories. Therefore, 46 male and 186 female respondents of those 9 RMG factories were invited to participate in this study by following simple random procedure from the list.

### Inclusion and exclusion criteria

**Fig 1:**
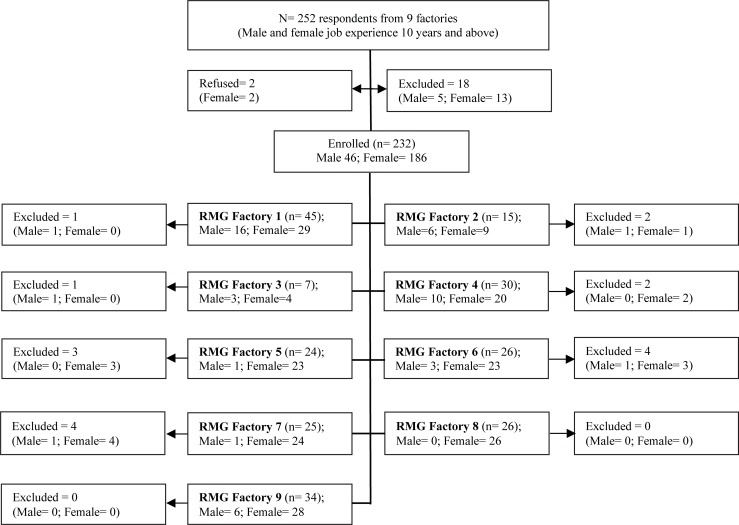
Recruitment flow chart.

The selection criteria were i) age of the respondents >18 years; ii) had at least 10 years of continuous work experience in RMG sector. Pregnant women and people with disabilities were excluded from the study. 18 individuals (5 males and 13 female) excluded from the study following exclusion criteria. Two female respondents refused to participate in this study (Fig 1).

### Data collection tool

The term WMSDs refers to work related injuries to bodily structures such as muscles, joints, tendons, ligaments, nerves, bones and the localized blood circulation system, caused or aggravated primarily by work itself or by the work environment [23]. The final questionnaire included three domains.

The *first domain* was designed for obtaining demographic information about age, gender, education, marital status, height, weight, body mass index (BMI), current occupation, years of experience, income, work days per week, work hours per day, breaks during work, and information regarding past WMSDs (injury history, injury regions, duration since onset, treatment history, and relapse situation). Asian BMI criteria were used for anthropometric measurement (height, weight) [24]. BMI was calculated by the formula weight/height squared (kg/m2) [24]. The *second domain* captured information on WMSDs, including prevalence by body regions, frequency of prevalence, effect of WMSDs on taking sick leaves, considering a job change, work performance, visit to health professionals and information on medication. Prevalence of WMSDs for each body region was determined by the Nordic Musculoskeletal Questionnaire-Extended (NMQ-E) tool. The investigation covered nine body regions: neck, shoulder, upper back, elbows, wrists/hands, lower back, hips/thighs, knee, and ankle/feet. The *third domain* contained information regarding work-related ergonomic risk factors and symptoms by the body regions. Quick Exposure Check (QEC) tool was applied for assessment of ergonomic risk factors of WMSDs. Ergonomic risk factors include force, lifting and carrying heavy loads, repetitive movements, awkward posture, duration of exposure, as well as environmental and psychosocial factors which increase the worker's chance of getting a WMSD [25].

NMQ-E and QEC were translated into Bangla (local language) and afterwards back translated into English by independent bilingual researchers and compared to the original English version by a third bilingual researcher. Finally, all three individuals discussed any disagreement and finalized the translated version. The Bengali version was pretested in the field and edited with input from the data collectors.

#### Nordic Musculoskeletal Questionnaire-Extended (NMQ-E)

Over two decades ago, Kuorinka and colleagues [26] presented the general Standardized Nordic Questionnaire as a screening instrument that comprised of just three questions regarding musculoskeletal pain that was widely utilized in the absence of any other rigorously reliable assessment tool. The NMQ-E tool used in this study has been adapted from Dawson et al. [27] which collects reliable information regarding the point, 12-month and lifetime prevalence, and consequences of musculoskeletal symptoms in nine body regions.

The NMQ-E has been previously applied to a wide range of occupational groups elsewhere to evaluate musculoskeletal problems, including computer and call center workers [28, 29], car drivers [30], coopers in the whisky industry [31], nursing [32] and forestry workers [33]. Previous studies concluded that the NMQ-E is repeatable, sensitive and useful as a screening and surveillance tool to measure the prevalence and repercussions of musculoskeletal pain and related events in studies of occupational and general populations [27, 34, 35].

**Fig 2:**
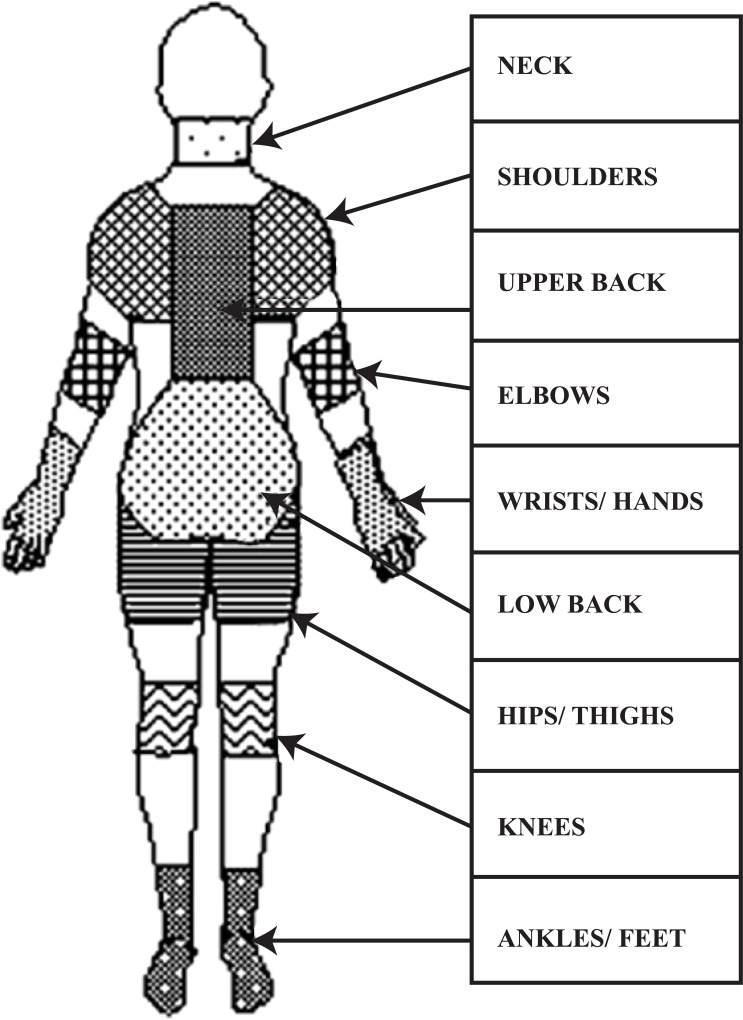
NMQ-E picture reproduced from Dawson et al. [27].

NMQ-E has a convenient one-page design that can be completed in approximately 10 to 15 minutes. In total, the NMQ-E comprised of 11 questions asked in reference to 9 body regions (Fig 2), equating to 99 data items generated by the tool. With the exception of age data, all response options are dichotomous (yes/no). 63 forced-choice items identifying areas of the body experiencing musculoskeletal problems. Completion is aided by a body map to indicate nine symptom sites- neck, shoulders, upper back, elbows, wrist/hands, lower back, hips/thighs, knees and ankles/feet. Respondents were asked if they had any musculoskeletal trouble 1) ever 2) duration of pain 3) hospitalization due to pain 4) change of job or duties due to pain 5) in the last 12 months 6) last month and 7) today which hampered normal activity. 36 forced-choice questions elicit 1) functional impact at home and work 2) assessment by a health professional 3) Medication taken and 4) taken sick leave during the last 12 months.

#### Ergonomics assessment: Quick Exposure Check (QEC)

Physical exposure to musculoskeletal risks was assessed with a pen-and-paper observation method called a Quick Exposure Check (QEC) [36]. QEC is a sensitive method for assessing physical exposure to musculoskeletal risks in the workplace with fair inter and intra-observer reliability [37].

*Step 1 (Observation by the evaluator and questions to the worker)*: The QEC is an instrument that assesses ergonomic risk factors, including physical, organizational and psychosocial factors. It is composed of an evaluation form that includes 16 questions about postures and movements performed by the spine and upper limbs, as well as other risk factors (amount of weight handled; how long it takes to perform a task; manual force; visual demand; vibration and level of hand force exerted; work pacing; and stress), and a score that allows for a partial (by body area) and total quantification of risk. This score results from the combination of answers given by the evaluator and the workers were calculated by entering the score, derived from each question.

*Step 2*: The calculated score was entered in to SPSS. The score was classified according to four categories of risk exposure: low, moderate, high, and very high (Table 1) [7, 36].

**Table 1:** Interpretation of the Quick Exposure Check scores.

Body area	Level of exposure			
	Low	Moderate	High	Very high
Back static	8–14	16–22	24–28	30–40
Back dynamic	10–20	22–30	32–40	42–56
Shoulder/Arm	10–20	22–30	32–40	42–56
Wrist/Hand	10–20	22–30	32–40	42–56
Neck	4–6	8–10	12–14	16–18
Vibration	1	4	9	0
Work space	1	4	9	0
Stress	1	4	9	0

Source: David et al. [7, 36]

*Step 3*: Finally, the sum of scores derived from four anatomic regions was divided by 176 and 162 for manual material handling and the other tasks, respectively.

*Step 4*: At last, the Action Levels (AL) of the WMSD was determined [36] as follows: AL 1 QEC Score ≤40% indicates acceptable musculoskeletal loading; AL 2 QEC Score 41%-50% indicates further investigation is needed and changes may be required; AL 3 QEC Score 51%-<70% indicates investigation and changes are required soon; AL 4 QEC Score >70% indicates investigation and change immediately are required.

### Training of the data collectors and pretesting

Six experienced data collectors (five women, one man) were recruited and trained for two weeks prior to commencing the study. They were graduates of health and social science disciplines. Training was provided by a physiotherapist and the research team. QEC training course was performed prior to data collection following the instruction from David et al. [7] that comprised background, using QEC effectively, practical trials, feedback, case studies and piloting. The NMQ-E and QEC were pilot-tested on a sample of RMG female participants (n = 20) in order to evaluate feasibility, time and cost. The main purpose of the pilot was also to identify any problems regarding the design and readability of the tool. Since the tool is utilized with a range of occupational populations (RMG), a secondary objective was to ensure that the instruments were interpretable by individuals with or without anatomical knowledge. Minor format modifications such as ‘Annual taken medication’ in the NMQ-E were made on the basis of this pilot prior to administering the survey. The data from the pilot study was not included in the main study. After piloting and essential modification, the tools were reviewed by study team and finalized.

### Data collection

NMQ-E Questions were ordered in such a way that those relating to the respondents’ lifetime (‘‘ever”) were asked first, followed by prevalence questions, and lastly items relating to consequences of pain in the previous year. Respondents were asked to answer all questions for a body region before progressing to the next region (i.e., horizontally rather than vertically). For each body region, if any respondent answered ‘no’ in 2 instances (specifically, questions relating to lifetime and annual prevalence of trouble), they were directed to go on to the next body region and all remaining questions for that region were automatically coded as negative responses. Lifetime symptom and 12 month prevalence has been used, as it is widely used in case of WMSD [38]. Severity of the symptoms was also measured by assessing the impact of the WMSDs on work activities. The interview using the questionnaire was conducted in a face to face. On an average, it took 40–50 minutes to complete the questionnaire. Data collection was done from 9am to 6pm on weekdays.

### Data analysis

Analysis was carried out using the IBM Statistical Package for Social Sciences (SPSS) Version 22 [39]. All the continuous variables were presented as mean and categorical data as percentage. Prevalence of WMSDs for each body region (neck, shoulders, upper back, elbows, wrist/hand, lower back, hips/thighs, knees and ankles/feet) was determined for the RMG workers. The results obtained from NMQ-E checklists was tested with QEC assessment results to find possible relationships. Descriptive statistics were carried out for all subjects to assess exposure risks and demographic information. The association between WMSDs factors and ergonomic parameters was analyzed using the chi-square (χ2) test. All P values presented are two-tailed and a *P*-value of <0.05 were considered as significant.

### Ethical approval

Ethical approval was obtained from the Ethical Review Committee of the James P Grant School of Public Health, BRAC University, Bangladesh. Permission to recruit the workers was obtained from the respective RMG factories. Written informed consent was obtained from each participant. Participants were compensated for their time. Interviews were conducted at a time and place, inside the factory premises, which was convenient to the participants.

## Results

Participants’ demographic characteristics are presented in Table 2. The mean age was 31.3 years (SD = 7). Majority of the participants were either without any formal schooling (28.9%) or had only primary education (44%) (p = 0.05). Among the total respondents, 49.6% were sewing operator (p = 0.001) and 95.7% respondents were doing overtime work. Around 40% of the respondents’ average monthly income, including overtime payment, was up to 15 thousand BDT (USD $190) with majority (59.5%) were earning between 15 and 20 thousand BDT (p = 0.05). Around 21% of the respondents were poor. In this study, majority (47%) were in the middle income quintiles. The mean weight, height, and Body Mass Index (BMI) were 55.09 kg (SD = 8.98), 1.53 m (SD = 0.08), and 23.51 kg/m^2^ (SD = 3.74) respectively (p = 0.05).

**Table 2:** Demographic characteristics of the respondents.

Characteristics		Male	Female	Total	P-value (χ2)
		n = 46	n = 186	N = 232	
**Age** Mean ± SD		**32.04 ± 7.3**	**31.17 ± 6.9**	**31.30 ± 7.0**	
	20–24 years	3 (6.5)	21 (11.3)	24 (10.3)	.874
	25–29 years	18 (39.1)	71 (38.2)	89 (38.4)	
	30–34 years	10 (21.7)	33 (17.7)	43 (18.5)	
	35–39 years	8 (17.4)	35 (18.8)	43 (18.5)	
	40 years and above	7 (15.2)	26 (14.0)	33 (14.2)	
**Education**					
	No formal education	7 (15.2)	60 (32.3)	67 (28.9)	.050*
	Up to primary level (1–5)	21 (45.7)	81 (43.5)	102 (44.0)	
	Up to secondary level (6–10)	18 (39.1)	43 (23.1)	61 (26.3)	
	Above secondary level	0 (0.0)	2 (1.1)	2 (0.9)	
**Marital status**					
	Married	36 (78.3)	136 (73.1)	172 (74.1)	.010*
	Unmarried	10 (21.7)	17 (9.1)	27 (11.6)	
	Widow	0 (0.0)	11 (5.9)	11 (4.7)	
	Divorced	0 (0.0)	5 (2.7)	5 (2.2)	
	Separated	0 (0.0)	17 (9.1)	17 (7.3)	
**Current occupation (designation)**					
	Sewing Operator	9 (19.6)	106 (57.0)	115 (49.6)	.000**
	Quality Inspector	2 (4.3)	21 (11.3)	23 (9.9)	
	Helper	1 (2.2)	17 (9.1)	18 (7.8)	
	Machine Operator	8 (17.4)	1 (0.5)	9 (3.9)	
	Others	26 (56.5)	41 (22.0)	67 (28.9)	
**Job experiences (years)**					
	10–15 years	38 (82.6)	170 (91.4)	208 (89.7)	.027*
	16–20 years	5 (10.9)	13 (7.0)	18 (7.8)	
	21–25 years	1 (2.2)	3 (1.6)	4 (1.7)	
	26 years and above	2 (4.3)	0 (0.0)	2 (0.9)	
**Own monthly income (BDT)** Mean ± SD				15186 ± 2322	
	<10000 (<126.5 USD)	2 (4.3)	8 (4.3)	10 (4.3)	.002*
	10001–15000 (126.50–189.75 USD)	8 (17.4)	74 (39.8)	82 (35.3)	
	15001–20000 (189.76–253.00 USD)	34 (73.9)	104 (55.9)	138 (59.5)	
	>20000 (>253.00 USD)	2 (4.3)	0 (0.0)	2 (0.9)	
**Socio-economic status** Mean ± SD				3.18 ± 0.94	
	Poorest	1 (2.2)	6 (3.2)	7 (3.0)	.165
	Second poorest	12 (26.1)	30 (16.1)	42 (18.1)	
	Middle income	16 (34.8)	93 (50.0)	109 (47.0)	
	Forth	14 (30.4)	37 (19.9)	51 (22.0)	
	Richest	3 (6.5)	20 (10.8)	23 (9.9)	
**BMI (kg/m**^**2**^**)** Mean ± SD		22.38 ± 3.2	23.79 ± 3.8	23.51 ± 3.7	
	Underweight (≤18.49kg/m2)	4 (8.7)	13 (7.0)	17 (7.3)	.013*
	Normal (18.50–24.99 kg/m2)	35 (76.1)	102 (54.8)	137 (59.1)	
	Overweight and obese (≥ 25.0 kg/m2)	7 (15.2)	71 (38.2)	78 (33.6)	

*Significant at p<0.05

**Significant at p<0.01

Others = Mending Operator, Assort Man, Check Man, Cutting Man, Folder, Iron Man, Labor, Measurement, Overlock Operator, Packing Man, Poly Man, Print Assistant, Quality Checker, Sample Man, Senior Cutting Man, Senior Operator, Senior Overlock Operator, Senior Printer, Senior Sewing Operator, Trimming Operator, Wash Man. 1 USD = 79.05 BDT (August: 2016)

### Prevalence of WMSDs

**Fig 3:**
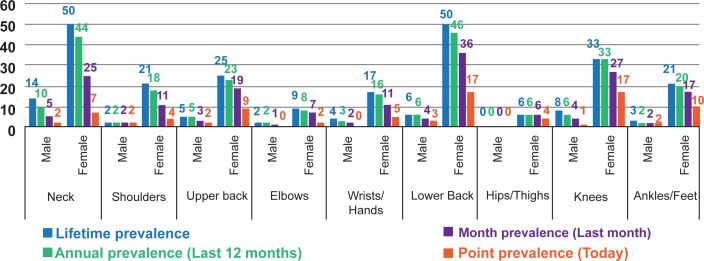
Prevalence of WMSDs in different body regions by the respondents.

**Fig 4:**
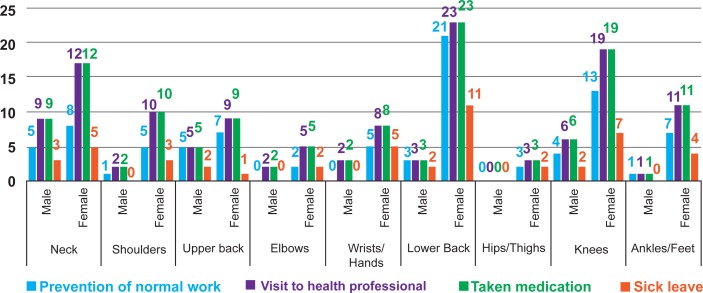
Consequences of WMSDs in different body regions by the respondents.

The prevalence of WMSDs differed considerably by gender. For female, the 12 months’ prevalence of WMSDs was highest in lower back (24.7%; n = 46), followed by neck (23.7%; n = 44) and knees (17.7%; n = 33). For male, the 12 months’ prevalence of WMSDs was highest in neck (21.7%; n = 10) followed by knees (13%; n = 6), lower back (13%; n = 6) and upper back (10.9%; n = 5) (Fig 3). Due to lower back pain, significant number of both male and female suffered from normal work activities, visited health professional, took medication and sick leave (Fig 4).

Among all the nine body regions, hips/thighs was the most frequently reported WMSD (Table 3) with the increase of age (37.8 ± 3.6), BMI (26.2 ± 2.4), total job experience (14.5 ± 7.0) and daily working hour (11.1 ± 0.4).

**Table 3:** Distribution of pain in different body region according to age, BMI, job experience and daily working hour.

Body region	Age (year)	BMI (Kg/M2)	Total job experience (year)	Daily working hour
	Mean ± SD	Mean ± SD	Mean ± SD	Mean ± SD
Neck	31.3 ± 6.6	23.3 ± 3.2	11.9 ± 2.9	11.1 ± 0.5
Shoulders	33.7 ± 9.7	24.2 ± 3.7	13.0 ± 5.1	11.1 ± 0.3
Upper back	32.1 ± 8.3	23.1 ± 3.2	12.6 ± 4.1	11.0 ± 0.4
Elbows	34.1 ± 7.5	23.4 ± 3.1	14.1 ± 5.2	10.8 ± 0.7
Wrists/hands	32.3 ± 5.9	25.5 ± 4.2	13.5 ± 4.2	11.1 ± 0.8
Lower back	31.7 ± 5.6	23.8 ± 3.3	12.1 ± 3.1	11.0 ± 0.6
Hips/Thighs	37.8 ± 3.6	26.2 ± 2.4	14.5 ± 7.0	11.1 ± 0.4
Knees	34.2 ± 6.1	24.3 ± 3.6	12.2 ± 3.3	10.9 ± 0.6
Ankles/feet	35.0 ± 7.1	24.6 ± 3.8	13.7 ± 6.4	11.1 ± 0.4

Table 4 presents the factors associated with twelve month WMSD’s in the different body region by age, BMI, job experience and daily working hour. Statistically significant relationship was found between WMSDs in elbows (p = 0.02), hips (p = 0.01), knees (p = 0.01) and ankle (p = 0.05) with age; upper back (p = 0.001), elbows (p = 0.001), wrists (p = 0.03), hips (p = 0.001) and ankles (p = 0.01) with job experience; hips with BMI (p = 0.03); elbows (p = 0.04) with daily working hour.

**Table 4:** Factor associated with 12-month prevalence of WMSDs in the different body region by age, BMI, job experience and daily working hour (n = 232).

Characteristics	Neck	Shoulders	Upper back	Elbows	Wrists/hands	Lower back	Hips/Thighs	Knees	Ankles/feet
	χ^2^ (p)	χ^2^ (p)	χ^2^ (p)	χ^2^ (p)	χ^2^ (p)	χ^2^ (p)	χ^2^ (p)	χ^2^ (p)	χ^2^ (p)
**Age**									
20–24 years 25–29 years 30–34 years 35–39 years 40 years and above	3.77 (0.44)	2.36 (0.66)	2.63 (0.62)	11.69 (0.02)*	2.29 (0.68)	6.29 (0.17)	13.34 (0.01)**	12.11 (0.01)**	9.15 (0.05)*
**Job experiences (years)**									
10–15 years 16–20 years 21–25 years 26 years and above	2.03 (0.56)	4.78 (0.18)	14.19 (0.00)**	18.63 (0.00)**	8.63 (0.03)*	2.13 (0.54)	36.58 (0.00)**	3.33 (0.34)	10.73 (0.01)**
**BMI (kg/m**^**2**^**)**									
Underweight Normal Overweight and obese	0.21 (0.90)	0.55 (0.75)	0.62 (0.73)	0.89 (0.64)	5.72 (0.05)	1.88 (0.39)	6.85 (0.03)*	3.69 (0.15)	5.09 (0.07)
**Daily working hour**									
0 to 10 hours > 10 hours	0.75 (0.38)	1.39 (0.23)	0.23 (0.62)	3.98 (0.04)*	0.00 (0.92)	0.00 (0.94)	0.33 (0.56)	0.46 (0.49)	0.54 (0.46)

*Significant at (p = 0.05) level

**Significant at (p = 0.01) level

Table 5 shows QEC risk scores categorized in low, medium, high and very high (based on Table 1) for different types of ergonomic factors. There was around 6 to 6.5% missing value for each type of QEC risk scores as observation was not allowed for 14 respondents of a RMG factory. Following risk exposure score in different body regions proposed by David et al. [7, 36] major respondents scored medium risk score of considering body region of wrist (male: 72.1%; female: 72%) followed by shoulder (male: 60.5%; female: 76.6%) and then Back-dynamic (male: 40.9%; female: 76.39). Medium score was obtained due to stress (male: 37.2%; female: 26.4%). Notably, very high risk was found to be highly prevalent in neck (male: 51.2%; female: 73.7%) only.

**Table 5:** Risk exposure score in different body regions (n = 218).

Risk rating	Low		Medium		High		Very high	
(RR)	M n (%)	F n (%)	M n (%)	F n (%)	M n (%)	F n (%)	M n (%)	F n (%)
**Back Static**	4 (20.0)	22 (16.2)	7 (35.0)	71 (52.2)	7 (35.0)	38 (27.9)	2 (10.0)	5 (3.7)
**Back dynamic**	9 (40.9)	9 (23.1)	9 (40.9)	30 (76.9)	4 (18.2)	0 (0.0)	-	-
**Shoulder**	2 (4.7)	18 (10.3)	26 (60.5)	134 (76.6)	12 (27.9)	23 (13.1)	3 (7.0)	0 (0.0)
**Wrist**	1 (2.3)	12 (6.9)	31 (72.1)	126 (72.0)	9 (20.9)	36 (20.6)	2 (4.7)	1 (0.6)
**Neck**	2 (4.7)	7 (4.0)	10 (23.3)	18 (10.3)	9 (20.9)	21 (12.0)	22 (51.2)	129 (73.7)
**Vibration**	20 (46.5)	120 (69.0)	2 (4.7)	1 (0.6)	21 (48.8)	53 (30.5)	-	-
**Work space**	40 (93.0)	150 (85.7)	3 (7.0)	25 (14.3)	-	-	-	-
**Stress**	27 (62.8)	124 (71.3)	16 (37.2)	46 (26.4)	-	4 (2.3)	-	-

M = Male; F = Female; Observation missing = 14 (restricted by the garment authority)

In Table 6, each case was interpreted in accordance to the Action Levels (AL). The results of the assessment of physical exposure to musculoskeletal risks by QEC technique showed that 9.2%, 8.7%, 77.5% and 4.6% of the studied workers was in Action Level 1 (AL1), AL2, AL3 and AL4 respectively *(P<0*.*003)*.

- in 9.2% of the RMG workers, the overall exposure level was less than 40% indicating that the level of exposure to WMSDs risks was acceptable (low risk).
- in 8.7% of the RMG workers, the overall exposure level was between 41% and 50% indicating that the level of exposure to WMSDs risks needed consideration (moderate risk).
- in 77.5% of the RMG workers, the overall exposure level was between 51% and 70% indicating that the level of exposure to WMSDs risks was high and ergonomics intervention to decrease exposure level seemed essential (high risk) and
- in 4.6% of the RMG workers, the overall exposure level was more than 70% indicating that the level of exposure to WMSDs risks was very high and investigation and change immediately are required (very high risk).

**Table 6:** The relationship between the prevalence rate of WMSDs within last 12-month period and risk level of WMSD achieved by QEC method (n = 218).

Risk Level (Overall Exposure)	Action level	WMSDs		
		Reported n (%)	Not Reported n (%)	Total n (%)
Low	AL1 (≤ 40%)	7 (35.0)	13 (65.0)	20 (9.2)*
Moderate	AL2 (41%-50%)	4 (21.1)	15 (78.9)	19 (8.7)*
High	AL3 (51%-70%)	95 (56.2)	74 (43.8)	169 (77.5)*
Very high	AL4 (>70%)	7 (70.0)	3 (30.0)	10 (4.6)*
Total		113 (51.8)	105 (48.2)	218 (100)

*Significant at (p<0.003) level (combined high and very high Action level for P value)

## Discussion

To our knowledge, this is the first study concerning prevalence of WMSDs in nine body regions and ergonomics assessment of their exposure to risk factors for the development of WMSDs among RMG worker’s in Bangladesh. Our participants were relatively older and experienced than other Bangladeshi studies [18, 19] due to our methodological variation and inclusion criteria. We found higher rate of primary level of education (44%) as compared with Labor Force Survey of Bangladesh (40.4%) [40].

Bangladesh is a labor-abundant developing country where very cheap price low-skilled workforce attracts foreign and local investors. We found, almost all of the workers were underpaid (earned up to 253 USD) which ultimately lower their life standard and increase their health vulnerabilities. “The Bangladesh Labour Law- 2006” (Act No. 42 of 2006) was passed by the parliament [41–43] and currently an Act adopted to amend (Act No. 30 of 2013) [44] further the Bangladesh Labour Act, 2006. Despite being a signatory member of the International labour Organization (ILO), the RMG sector in Bangladesh underway though in slow pace to ensure ILO conventions such as eight hours work, weekly holiday and minimum wages [45, 46]. Interestingly, due to pressure by the buyers, reputable RMG factory owners of Bangladesh are now trying to improve the quality of the health and safety of the workers [20].

In this study, we found greater number of respondents (around 34%) who were overweight and obese (BMI ≥25 kg/m2). Our findings was higher than previous studies from Bangladesh [47–49]. This might be due to inclusion of experienced and relatively aged workers in our study. In addition, around 50% of our study population were sewing operator where they sit or stand for extended periods of time with minimum body movement. This might be linked with overweight and obesity. This is consistent with previous study where six of every ten respondents are suffering from musculoskeletal disorder among the Bangladeshi RMG workers that is associated with body weight [19]. Our mean BMI (23.51 ± 3.7) was higher than such workers in Bangladesh (22.9±3.4) [50] and Sri Lanka (20.9 ± 3.6) [51].

Among female respondents in our study, around 25% reported lower back pain (overall 22.41%) that was higher than previous study from Bangladesh (18%) [52]. Our prevalence rate of lower back pain was consistent with nationwide study of Taiwan [13] and China [53]. This is in line with the findings among 210 workers of thirty five garment factories from Jaipur, India [54]. Earlier empirical evidence also suggest that RMG workers suffer from WMSDs, particularly of neck and back regions are the most frequently reported [55]. In addition to back and neck pain, WMSDs of other body parts were also prevalent among workers. In India, WMSDs are accounted for 78.5% of all work related illness and disorders [56]. Evidence showed that use of short breaks can minimize the risk and occurrence of low back pain [57].

Findings of our study showed statistically significant relationship between twelve month WMSD’s in different anatomical region with age, BMI, total job experience and daily working hours. Earlier handful studies [17, 19] reported gender, age, length of service, nature and posture of work were significantly associated with WMSDs that is also related to our study. These findings are similar to a study among the Iranian sewing machine operators of a shoe manufacturing factory [58].

Previous researches showed that workers who have been employed for longer period of time had less chance to encounter with occupational injuries than recently employed workers [59, 60]. Workers with lower job duration did not have enough experience to meet ergonomic risk factors because this situation had impacts on their interactions with workplaces.

Following risk exposure score in different body regions, a significant number of respondents scored medium risk score of considering body region of shoulder and wrist. These risk factors resulted in musculoskeletal complaints, sick leave, and switching and leave jobs. QEC assessment showed that level of exposure to musculoskeletal risks was high among 80% of our study population that was aligned with the evident that ergonomically the garment factories were poor in Bangladesh [17]. This finding indicated that the nature of jobs and working conditions in the garment factories was conducive for developing WMSDs. Therefore, ergonomic interventions and corrective measures are necessary to improve the working conditions and decrease the exposure level to musculoskeletal disorders. One aspect of improving worker’s health in garment factories includes addressing WMSDs risk factors through ergonomic interventions [20], a low cost solution that was successfully implemented previously in Bangladesh [18]. Evidence also showed that exercises in the work place are effective in reducing the severity from low back pain [61].

In our study, we used combination of observation and structured questionnaire like NMQ-E and QEC for Ergonomic assessment to generate data that might be useful in improving the ergonomic and comfort of RMG workers’ workplaces in Bangladesh. This has not been studied previously. Furthermore, the prevalence of WMSDs among workers followed two main purposes: detecting musculoskeletal disorders’ prevalence rate and finding causative and other relative factors which had impact on this rate.

The findings of this study are subject to some limitations. First, this cross-sectional study does not show any causality between WMSDs factors and ergonomic parameters. This study inspires future studies. Second, we did not apply any measurement scale for measuring the intensity of the pain/discomfort which was reported by respondents. The experience of the person who complete the questionnaire may affect the results. Recent and more serious WMSDs are prone to be remembered better than older and less serious ones. The environment and filling out situation at the time of the questioning may also affect the results. The life time prevalence of musculoskeletal symptom and its related questions might provide high probability of recall bias. Previous studies show that a longer recall period (12 month) is likely to cause recall bias regarding injuries, especially if the injuries are less severe [62, 63]. The QEC emphasizes posture and doesn't provide much information about force or recovery time, which are important factors of WMSDs. From an epidemiological viewpoint, it is evident that this type of questionnaire is most applicable for cross-sectional studies with all the concomitant limitations. One of the problems of a cross-section study is dropout of affected workers so that the study group may be affected by "the healthy worker effect" and only includes the survivors. The study population is representative of the proportion of population of workers in urban RMG sector of Bangladesh who have more than ten years of work experiences.

## Conclusions

This study revealed the 12-month prevalence of WMSDs symptoms among studied RMG workers was high and females were affected more. Specifically, the lower back, neck and knees were mostly affected areas. Moreover, QEC findings revealed that, level of physical exposure to WMSDs risks is high among eighty percent of the RMG workers and ergonomic intervention along with investigation and change to decrease exposure level is essential. The ergonomic factors in terms of the work pace, awkward posture, seat, hand position during work, repetitive movement and stress which might lead to many musculoskeletal disorders amongst the workers. In general, a low cost solution can be recommended for policy makers to reduce exposure to musculoskeletal risks. These include using work rest cycle, chair with backrests, hand position during work, floor mats for standing tasks (e.g. cutting), tilting the worktables by using wooden wedges under the legs, implementing training programs with work safety awareness, and playing background music.

## Supporting information

S1 Fig Development and test–retest reliability of an extended version of the Nordic Musculoskeletal Questionnaire (NMQ-E): A Screening instrument for musculoskeletal pain. (PDF)

S1 File Ethical Review Committee (ERC) approval letter. (PDF)

S2 File Dataset. (SAV)

## Abbreviations

AL: Action Level
BDT: Bangladesh Taka
BGMEA: Bangladesh Garment Manufacturers and Exporters Association
BLL: Bangladesh Labour Law
BMI: Body Mass Index
CRP: Centre for Rehabilitation of the Paralysed, Bangladesh
DALYs: Disability-adjusted life-years
ILO: International Labour Organization
LBP: Low back pain
MSD: Musculoskeletal disorder
NIOSH: National Institute for Occupational Safety and Health
NMQ-E: Nordic Musculoskeletal Questionnaire-Extended
QEC: Quick Exposure Check
RMG: Ready Made Garment
RR: Risk rating
SD: Standard deviation
SPSS: IBM Statistical Package for the Social Sciences
USD: United States Dollar
WHO: World Health Organization
WMSD: Work-related musculoskeletal disorder
YLDs: Years lived with disability
