# Supplementary material for: Prevalence of work related musculoskeletal disorders (WMSDs) and ergonomic risk assessment among readymade garment workers of Bangladesh: A cross sectional study
Source: PLoS One. 2018 Jul 6;13(7):e0200122. doi: 10.1371/journal.pone.0200122 (PMC6034848; doi:10.1371/journal.pone.0200122)
Supplement: S1 Fig — (PDF) [file pone.0200122.s001.pdf]

The Journal of Pain, Vol 10, No 5 (May), 2009: pp 517-526
Available online at www.sciencedirect.com

ELSEVIER

Development and Test–Retest Reliability of an Extended Version of the Nordic Musculoskeletal Questionnaire (NMQ-E): A Screening Instrument for Musculoskeletal Pain

Anna P. Dawson,* Emily J. Steele,† Paul W. Hodges,* and Simon Stewart¶

**NHMRC Centre for Clinical Research Excellence in Spinal Pain Injury and Health, The University of Queensland, Brisbane, Australia.*
†Discipline of Public Health, University of Adelaide, Adelaide, Australia.
¶Baker Heart Research Institute, Melbourne, Australia.

Abstract: The Nordic Musculoskeletal Questionnaire (NMQ) quantifies musculoskeletal pain and activity prevention in 9 body regions. The purpose of this study was to develop an extended NMQ (NMQ-E) to collect greater information regarding musculoskeletal pain, examine test–retest reliability and the reproducibility of alternate administration methods. Reliability was examined using observed proportion of agreement for all (P_o), positive (P_{pos}) and negative (P_{neg}) responses, kappa (κ), proportion of maximum kappa achieved (κ/κ_{max}), intra-class correlation coefficient (ICC) and standard error of measurement (SEM). The NMQ-E was self-administered by 59 Bachelor of Nursing students at a 24-h interval with mean P_o = 0.88–0.98 and κ/κ_{max} = 0.71–0.96 for 10 dichotomous questions and mean $ICC_{(2,1)}$ = 0.97 and SEM = 1.05 years for the age at symptom onset question. The NMQ-E was completed via self and interview administration by 31 student nurses at a 0.97 ± 1.14 day interval with mean P_o = 0.92–0.98 and κ/κ_{max} = 0.76–1.00 for binary questions and mean $ICC_{(2,1)}$ = 0.90 and SEM = 1.51 years for age at symptom onset data. In both sub-studies, mean P_{pos} was lower than mean P_{neg} and low prevalence reduced κ in many instances. The NMQ-E collects reliable information regarding the onset, prevalence, and consequences of musculoskeletal pain and can be administered by self-completion and personal interview.

Perspective:* *This study presents an NMQ-E that collects reliable information regarding the onset, prevalence, and consequences of musculoskeletal pain in 9 body regions. The NMQ-E can be utilized in descriptive studies or longitudinal studies of disease outcome and can be administered via self-completion and personal interview.

© 2009 by the American Pain Society

Key words: *Nordic Musculoskeletal Questionnaire, musculoskeletal pain, questionnaire, reliability.*

A cornerstone of epidemiological investigations of musculoskeletal pain is the collection of reliable data. Two decades ago, Kuorinka et al[25] presented the general Standardised Nordic Questionnaire as a screening instrument to quantify musculoskeletal pain and related activity prevention. The tool is most frequently referred to as the Nordic Musculoskeletal Questionnaire (NMQ), a more descriptive term introduced by Dickinson et al.[16] The NMQ has been used most extensively with occupational populations[7,9,17,29,37,40] and to a lesser degree with general populations.[20,32,42] Surprisingly, the literature chronology for the NMQ highlights that the instrument has been widely utilized in the absence of rigorous reliability assessment.

The reliability of questionnaires, a property also described as reproducibility, is the degree to which repeated measurements in stable study objects provide similar results.[14] The most rudimentary estimate of reliability is the proportion of observed agreement or disagreement.[19,38] Although it is a straightforward approach, if the condition assessed is very common or very rare, it is possible to get high agreement due to

Received May 11, 2008; Revised October 24, 2008; Accepted November 24, 2008.

Supported in part by the University of South Australia. Anna Dawson is supported by The Sir Robert Menzies Memorial Foundation Limited. Emily Steele, Paul Hodges and Simon Stewart are supported by the National Health and Medical Research Council of Australia. There are no conflicts of interest.

Address reprint requests to Anna Dawson, NHMRC Centre for Clinical Research Excellence in Spinal Pain Injury and Health, School of Health and Rehabilitation Sciences, The University of Queensland, St Lucia, QLD, 4072, Australia. E-mail: a.dawson1@uq.edu.au

1526-5900/$36.00

© 2009 by the American Pain Society

doi:10.1016/j.jpain.2008.11.008

chance alone.[38] When the NMQ was introduced over 20 years ago, only percentage disagreement data was available to attest to the reliability of the tool.[25] Despite this, uptake was extensive and the instrument gained acceptance. Studies that presented further reliability coefficients for the NMQ were not reported until 16 or more years after the tool was first published[5,13] and utilized the kappa (κ)[11] statistic in isolation despite warnings of problems associated with its use.[10,19] The κ statistic is a reliability coefficient developed to provide a chance-corrected estimate of agreement.[30] κ is affected by prevalence and data distribution and can produce misleading reliability statistics.[19] The reliability studies that used κ[5,13] also partly or wholly comprised patient samples although the tool was originally designed for occupational health research.[25] Since questionnaire reliability is specific to the respondent population in which it is examined, the reliability of NMQ responses should be established in an occupational cohort.

Instruments can be administered in a variety of forms such as self-completion (on paper or via the internet), telephone and personal interview. The responses obtained from different completion methods can differ,[4,15] so the reliability of specific administration techniques should be determined. Due to the limitations of κ, it is recommended to calculate a range of reliability coefficients in conjunction with the statistic. These include the proportion of positive and negative agreement,[10] and the proportion of maximum κ obtained.[36] Such data has not previously been determined for the NMQ.

The NMQ is comprised of just 3 questions regarding musculoskeletal pain including annual and 7-day prevalence of symptoms and annual prevention from normal work (at home or away from home). Studies frequently report modifying or adapting the NMQ,[7,23,26,31,40] which may be due to the limited data it collects in its original form. The first aim of this study was to develop an extended version of the NMQ (NMQ-E) to generate greater data regarding the prevalence and repercussions of musculoskeletal pain. The second objective was to determine test–retest reliability in an occupational cohort using a comprehensive range of reliability indices. The third aim of this study was to assess the reproducibility of data obtained via alternate administration methods.

Methods

Survey Development

The goal of this extended English language version of the NMQ was to generate more comprehensive data about musculoskeletal pain and related consequences while maintaining an economical single-page design. Questions taken from the location-specific Standardised Nordic Questionnaires (that is, the Low Back Questionnaire and the Neck and Shoulders Questionnaire)[25] were added to those of the general questionnaire as well as additional questions regarding annual medication usage and sick leave, taken from a previous study,[2] and the age of respondents at the onset of their symptoms. The instrument is outlined in Appendix 1. Further information can be obtained regarding the source of each NMQ-E question by contacting the authors. As in the original version,[25] the NMQ-E inquires about "trouble," defined as "ache, pain or discomfort" and 9 body regions (3 each on the upper limbs, spine and lower limbs) are visually depicted on a body chart viewed from behind. In total, the NMQ-E is comprised of 11 questions asked in reference to 9 body regions, equating to 99 data items generated by the tool. With the exception of age data, all response options are dichotomous (yes/no).

Questions were ordered in such a way that those relating to the respondents' lifetime ("ever") were asked first, followed by prevalence questions, and lastly items relating to consequences of pain in the previous year. Key words were highlighted in bold, as was done in previous versions.[16,25] Respondents were asked to answer all questions for a body region before progressing to the next region (ie, horizontally rather than vertically). In 2 instances (specifically, questions relating to lifetime and annual prevalence of trouble), if the respondent answered no, they were directed to go on to the next body region and all remaining questions for that region were automatically coded as negative responses.

The NMQ-E was pilot-tested on a sample that comprised undergraduate and postgraduate students from health and social science disciplines and academic staff employed at a university (n = 7). As questions were compiled largely from existing instruments, the main purpose of the pilot was to identify any problems regarding the design and readability of the tool. Since the tool is utilized with a range of occupational populations, a secondary objective was to ensure that the instrument was interpretable by individuals with and without anatomical knowledge. Pilot study participants were interviewed by the first author after completing the questionnaire to discuss whether issues arose. Minor format modifications were made on the basis of this pilot prior to administering the survey to the study cohort.

Test–Retest Reliability and Reproducibility of Administration Methods

Sample and Data Collection

As the NMQ was originally designed for occupational health research[25] and has frequently been utilized in nurse populations,[2,8,21-24,26,31,37,40] a nursing sample was selected for this study. University students enrolled in a Bachelor of Nursing degree program were considered appropriate as they were known to comprise a good proportion of mature aged individuals with previous workforce exposure and were more accessible than nurses in the workforce for fulfilling the requirements of a test–retest study. The reliability and reproducibility studies were undertaken in conjunction with a cross-sectional prevalence study of musculoskeletal disorders and related disability. Nursing students were provided with information about the study and invited to participate during lectures and tutorials at 1 rural and 2

metropolitan university campuses. Of the 460 individuals invited, 373 volunteered for the study, equating to a participation rate of 81%. As university administrators wished to minimize the impact of the study on students and the curriculum, it was not possible to access all students on 2 occasions to fulfil the requirements of a test–retest study. Rather, a convenience sample of volunteers from this cohort was utilized for the reliability studies. Test–retest reliability was assessed with the questionnaire completed twice by self-administration at a 24-h interval at the beginning or end of class time. The reproducibility of administration strategies was investigated focusing on 2 completion methods: Self-administration and face-to-face interview. This is a means of assessing the validity of self-reported information. Interviews were conducted by the first and second authors, who were qualified physiotherapists and thus experienced in inquiring about musculoskeletal pain and related consequences. The time interval between the 2 administrations was difficult to control due to participant availability but it was ensured that all interviews were conducted within 3 days of self-completion of the tool. A $5 voucher for a local café was provided in return for participation as interviews were conducted in the participant's personal time. The study was approved by the Institutional Human Research Ethics Committee and written consent was gained prior to participation.

Statistical Analysis

The reliability of dichotomous data produced by the NMQ-E was assessed using a range of indices including proportion of observed agreement (P_o),[19] and the κ coefficient.[11] The κ coefficient was calculated using $\kappa = (P_o - P_e)/(1 - P_e)$ where P_e is the proportion of agreement expected by chance.[19,30] A κ of 1.0 represents perfect agreement,[11] and a κ that is negative or zero reflects that the agreement between testing occasions was less than or equal to that expected due to chance, respectively.[41] As discussed, the coefficient κ as a single index of reliability has been shown to be unstable. Two paradoxes of κ have been described. First, when vertical and horizontal marginal totals of 2 $\times$ 2 test–retest convergence tables are symmetrically unbalanced, it is possible to get low κ even though there is high agreement. Second, κ can be inflated if marginal aggregates are asymmetrical rather than symmetrical, or imperfect as compared with perfectly symmetrical.[19] Hence, presenting the coefficient in conjunction with other indices provides greater insight about reliability. Observed proportion of positive agreement (P_{pos}) and negative agreement (P_{neg}) were calculated because they can highlight the sources of disagreement that a single statistic cannot express.[10] The maximum kappa obtainable (κ_{max})[11] and the proportion of κ_{max} achieved (κ/κ_{max})[18] were also determined. κ_{max} reflects the extent to which agreement between test and retest is constrained by marginal imbalances and replaces 1.0 as the maximum possible level of agreement that could be produced by the data.[36] κ/κ_{max} thereby represents the proportion of chance-corrected agreement attained in consideration of data limitations. Where κ/κ_{max} was equal to 0/0, a value of 1.0 (representing that the maximum κ was achieved) was assigned for mean calculations. The question regarding age of onset of symptoms was assessed using the intra-class correlation coefficient (ICC).[35] The reliability of test–retest designs are best analyzed using 2-way ICC models,[43] and in this study models with random effects, single measures and absolute agreement ($ICC_{[2,1]}$) were used in order to include both systematic and random error. The results of fixed effects models (that consider only random error) were contrasted with these findings to identify any systematic differences between testing occasions. Such differences in age data were also examined using the Wilcoxon signed rank sum test. The standard error of measurement (SEM) was calculated using the method outlined by de Vet et al[14] appropriate for absolute agreement ICC models: $SEM_{agreeement} = \sqrt{(\sigma^2_{test\text{-}retest} + \sigma^2_{residual})}$. The variance ($\sigma^2$) components for this calculation were determined using restricted maximum likelihood estimation.[14] Statistical analyses were performed in SPSS version 15.0 (SPSS Inc, Chicago, IL). Kappa coefficients were calculated by hand where SPSS could not compute a statistic due to data distribution.

Results

Test–Retest Reliability

The NMQ-E was twice self-administered at a 24-h interval by 59 student nurses. The personal characteristics of the sample (Reliability cohort) are presented in Table 1. The sample ranged in age from 18 to 51 years, with 27% aged >25 years. Sixty-four percent of the cohort

Table 1. Sample Characteristics

	Reliability Cohort	Reproducibility Cohort	Individuals in Both Reliability and Reproducibility Cohort
Sample (n)	59	31	7
Age (years)	23.4 ± 7.8	23.8 ± 10.0	26.6 ± 11.8
Weight (kg)	67.6 ± 12.0	69.2 ± 11.9	66.9 ± 11.8
Height (cm)	166.4 ± 7.5	167.7 ± 9.0	166.8 ± 8.2
% female	87.9	83.9	85.7
Prevalence of musculoskeletal trouble in the last 12 months* (%)			
Neck	49.2	44	14.3
Shoulder	46.7	23.7	28.6
Upper back	32.5	21.7	21.5
Elbow	2.6	0	0
Wrist/Hand	23.1	20.4	28.6
Low back	56.4	70	71.4
Hip/Thigh	13.6	18.4	28.6
Knee	32.2	25	42.9
Ankle/Foot	31.4	28.3	35.8

*Mean of 2 testing occasions.

Table 2. Test–Retest Reliability of the NMQ-E in an Occupational Cohort (n = 59): Age of Onset and Prevalence Questions

	Age at Onset of Trouble		Lifetime Prevalence			Annual Prevalence			Month Prevalence			Point Prevalence		
	ICC$_{(2,1)}$ (95% CI)	SEM (yrs)	κ	κ/κ_{max}	P_o	κ	κ/κ_{max}	P_o	κ	κ/κ_{max}	P_o	κ	κ/κ_{max}	P_o
Neck	0.98 (0.96–0.99)	0.49	0.85	0.92	0.93	0.66	0.77	0.83	0.81	0.92	0.92	0.69	0.85	0.91
Shoulders	0.97 (0.93–0.99)	1.11	0.79	0.79	0.90	0.76	0.85	0.88	0.71	0.81	0.88	0.91	1.00	0.98
Upper back	0.87 (0.70–0.95)	1.28	0.89	1.00	0.95	0.72	0.75	0.88	0.68	0.71	0.88	0.40	0.45	0.92
Elbows	1.00 (0.99–1.00)	0.29	1.00	1.00	1.00	1.00	1.00	1.00	0.00	0/0	0.97	0.00	0/0	0.98
Wrists/hands	0.98 (0.96–0.99)	0.91	0.84	0.91	0.93	0.76	0.80	0.91	0.57	0.80	0.90	0.00	0/0	0.97
Low back	0.98 (0.96–0.99)	0.69	0.81	0.91	0.92	0.69	0.77	0.84	0.70	0.76	0.86	0.34	0.34	0.82
Hips/thighs	0.98 (0.96–0.99)	1.48	0.83	0.88	0.95	0.71	0.71	0.93	0.55	1.00	0.95	0.00	0/0	0.98
Knees	0.98 (0.96–0.99)	1.34	0.93	1.00	0.97	0.61	0.66	0.83	0.71	0.71	0.93	−0.02	−0.03	0.95
Ankles/feet	0.97 (0.93–0.99)	1.84	0.90	0.93	0.95	0.65	0.67	0.85	0.76	0.80	0.92	0.68	0.81	0.93
Mean	0.97 (0.94–0.99)	1.05	0.87	0.93	0.94	0.73	0.78	0.88	0.61	0.83	0.91	0.33	0.71	0.94

Abbreviations: NMQ-E, Nordic Musculoskeletal Questionnaire (Extended version); ICC$_{(2,1)}$, intraclass correlation coefficient (two-way random model, single measures, absolute agreement); 95%CI, 95% confidence interval; SEM, standard error of measurement, κ, kappa coefficient; κ/κ_{max}, the proportion of maximum kappa achieved; P_o, proportion of observed agreement.

had previously worked in a position that involved manual work (including frequent or heavy lifting, frequent bending or twisting of the spine, or patient handling) and 17% had been employed as a caregiver, enrolled nurse or nurse's aide.

Reliability statistics for prevalence questions and the age at onset of symptoms are presented in Table 2, with means provided for each question comprising data from all 9 body regions. Lifetime prevalence demonstrated the greatest reliability whereas point prevalence displayed the least reliability (as we would expect). The question regarding age of onset of trouble had high mean ICC and there were no significant differences between test and retest for any body region (all $P \geq .14$) confirmed by identical estimates from fixed and random effects ICC models.

Reliability indices for questions inquiring about the repercussions of musculoskeletal pain are outlined in Table 3. There were 7 items where κ and P_{pos} could not be computed as responses were 100% negative on both testing occasions, resulting in a denominator of 0 in the respective calculations. Consequently κ/κ_{max} could not be calculated in these instances.

Regarding data for all binary questions on the NMQ-E, κ was negative in 2 instances and equal to 0 in 13 instances. These 15 questions had a corresponding mean P_o = .97 (range 0.91–0.98), which indicates there were few divergent responses corresponding with these low scores. In each case there were no individuals who reported yes on both testing occasions illustrated by P_{pos} = .00. For the 13 questions where κ = .00, κ_{max} also equaled 0, necessitated by marginal distributions in the fourfold concordance table, and thus the maximum value of κ was achieved.

The maximum percentage of disagreement for any question was 17%. Nearly half (45%) of all questions achieved the maximum κ obtainable given data distributions and 82% achieved κ/κ_{max}>0.70. There were 8 instances where questions scored κ/κ_{max}<0.50, including the point prevalence question for 3 body regions and 5 others scattered over a range of body regions and questions. This suggests that other than point prevalence, no particular question was less reliable. Mean P_{pos} ranged from .30 to .93 compared with .90 to .99 for mean P_{neg}, suggesting that the proportion of agreement was more stable for negative than positive answers. Data for P_{pos} and P_{neg} can be obtained from the authors for each dichotomous NMQ-E question upon request.

Reproducibility of Self-Administration and Interview Administration

Thirty-one participants completed the questionnaires by self-administration and face-to-face interview (Reproducibility cohort). Seven subjects who participated in this component of the study were also members of the Reliability cohort. Table 1 describes their characteristics. The sample ranged in age from 17 to 51 years with close to a quarter (23%) over 25 years of age. Greater than half (58%) of the participants had been previously employed

Table 3. Test–Retest Reliability of the NMQ-E in an Occupational Cohort (n = 59): Questions Regarding Consequences of Pain

	Lifetime Hospitalization		Lifetime Changed Jobs Or Duties		Annual Prevention Of Normal Work		Annual Visit To Health Professional		Annual Medication		Annual Sick Leave	
	κ / κ/κ_{max}	P_o	κ / κ/κ_{max}	P_o	κ / κ/κ_{max}	P_o	κ / κ/κ_{max}	P_o	κ / κ/κ_{max}	P_o	κ / κ/κ_{max}	P_o
Neck	1.00	1.00	0.64	0.95	0.47	0.93	0.86	0.95	0.45	0.90	0.66	0.98
	1.00		1.00		1.00		0.90		0.71		1.00	
Shoulders	1.00	1.00	0.54	0.95	0.85	0.98	0.83	0.93	0.47	0.93	0.00	0.98
	1.00		0.64		1.00		0.83		1.00		0/0	
Upper back	*	1.00	0.37	0.95	0.00	0.92	0.70	0.90	0.48	0.97	0.00	0.98
	*		0.47		0/0		0.70		0.48		0/0	
Elbows	1.00	1.00	*	1.00	*	1.00	*	1.00	*	1.00	*	1.00
	1.00		*		*		*		*		*	
Wrists/hands	−0.03	0.91	0.88	0.98	0.85	0.98	0.64	0.95	0.73	0.97	0.85	0.98
	−0.05		1.00		1.00		0.73		1.00		1.00	
Low back	0.00	0.98	0.48	0.88	0.48	0.88	0.76	0.89	0.66	0.91	0.00	0.95
	0/0		1.00		1.00		0.82		0.83		0/0	
Hips/thighs	0.00	0.98	0.00	0.98	0.00	0.98	0.46	0.93	0.85	0.98	*	1.00
	0/0		0/0		0/0		0.46		1.00		*	
Knees	0.79	0.98	1.00	1.00	0.31	0.93	0.70	0.95	0.30	0.93	0.00	0.98
	1.00		1.00		1.00		0.78		0.46		0/0	
Ankles/feet	0.81	0.95	0.44	0.90	0.62	0.91	0.83	0.95	0.40	0.91	0.54	0.95
	0.87		0.55		0.67		0.88		0.63		0.64	
Mean	0.57	0.98	0.55	0.95	0.45	0.95	0.72	0.94	0.54	0.94	0.29	0.98
	0.85		0.83		0.96		0.76		0.76		0.95	

*A kappa coefficient and κ/κ_{max} could not be calculated.

Abbreviations: NMQ-E, Nordic Musculoskeletal Questionnaire (Extended version); κ, kappa coefficient; κ/κ_{max}, the proportion of maximum kappa achieved; P_o, proportion of observed agreement.

in a role that involved manual work and 13% had worked in a caregiver or nursing position. The intervening interval between administrations was .97 ± 1.14 days (range 0–3 days), with 55% of the sample completing both administrations on the same day.

Reliability indices for the age at onset and prevalence questions are outlined in Table 4. There were no systematic differences for the age at onset of symptoms question between self- and interview administrations of the instrument ($P \geq .06$), and identical ICC were obtained from fixed and random effects ICC models. Data for questions concerning the consequences of musculoskeletal pain are presented in Table 5. Questions relating to hospitalization, medication usage, sick leave and so on displayed high mean κ/κ_{max} concurrent with high mean P_o and low mean κ, suggesting that low prevalence of positive responses had affected κ scores for some phenomena.

In consideration of all dichotomous questions on the NMQ-E, there was a minimum mean κ/κ_{max} of .76. Seventy-two percent of questions attained the maximum score of κ obtainable, and 87% scored $\kappa/\kappa_{max} \geq .70$. There were 19 instances where κ, κ/κ_{max} and P_{pos} could not be calculated, because responses were 100% negative on both testing occasions. Six questions had κ/κ_{max} values of less than .50, including the point prevalence question for 3 body regions. Mean P_{pos} ranged from .17 to .95 and mean P_{neg} varied from .92 to .99, indicating greater proportional stability for negative responses.

Discussion

This study was needed as the reliability of the NMQ has never been rigorously examined with a comprehensive range of coefficients in an occupational cohort, the population for which it was originally designed. Development of the NMQ-E was considered important because the original NMQ collects minimal data regarding musculoskeletal pain and activity prevention. For 10 dichotomous questions in the NMQ-E, mean proportion of agreement statistics indicate that the frequency of divergent responses was low. Kappa values were reduced for some questions by low frequencies of positive responses evident in our sample. Mean κ/κ_{max} data suggested that the extent of chance-corrected agreement obtained for dichotomous questions was 71% to 96% of the maximum possible score. The age at onset of symptoms question exhibited high reliability, with mean $ICC_{(2,1)} = .97$ (95% CI .94–.99) reflecting that ~97% of the observed score variance was attributable to true score variance and 3% was due to error.[39,43] According to the categories of κ described by Landis and Koch,[27] 77% of questions in the NMQ-E exhibited almost perfect, substantial or moderate reliability. However, given the widely recognized

Table 4. Reliability Coefficients for Self and Interview Administration of the NMQ-E in an Occupational Cohort (n = 31): Age of Onset and Prevalence Questions

	Age at Onset of Trouble		Lifetime Prevalence		Annual Prevalence		Month Prevalence		Point Prevalence	
			κ / κ/κ_{max}	P_o	κ / κ/κ_{max}	P_o	κ / κ/κ_{max}	P_o	κ / κ/κ_{max}	P_o
	$ICC_{(2,1)}$ (95% CI)	SEM (yrs)								
Neck	0.52 (0.03-0.81)	3.05	0.80<br>1.00	0.90	0.65<br>0.82	0.83	0.74<br>1.00	0.90	0.78<br>1.00	0.97
Shoulders	0.98 (0.90-0.99)	0.78	0.86<br>1.00	0.93	0.63<br>0.77	1.00	0.61<br>1.00	1.00	1.00<br>1.00	1.00
Upper Back	0.95 (0.67-0.99)	0.67	0.84<br>1.00	0.93	0.52<br>0.73	0.83	0.67<br>1.00	0.90	0.35<br>0.44	0.90
Elbows	0.99 (0.78-1.0)	0.58	1.00<br>1.00	1.00	*<br>*	1.00	*<br>*	1.00	*<br>*	1.00
Wrists/hands	0.71 (0.19-0.93)	2.45	1.00<br>1.00	1.00	0.67<br>1.00	0.90	0.47<br>1.00	0.93	*<br>*	1.00
Low Back	0.99 (0.98-1.0)	0.75	0.81<br>1.00	0.93	0.84<br>0.84	0.93	0.66<br>0.71	0.83	0.35<br>0.40	0.83
Hips/thighs	0.97 (0.85-0.99)	2.13	0.92<br>1.00	0.97	0.89<br>1.00	0.97	0.63<br>1.00	0.93	0.00<br>0/0	0.97
Knees	0.99 (0.99-1.0)	0.82	0.93<br>1.00	0.97	0.73<br>0.81	0.90	0.76<br>1.00	0.93	0.00<br>0/0	0.97
Ankles/feet	0.97 (0.91-0.99)	2.35	1.00<br>1.00	1.00	0.75<br>0.82	0.90	0.87<br>1.00	0.97	0.46<br>0.46	0.93
Mean	0.90 (0.57-1.00)	1.51	0.91<br>1.00	0.96	0.71<br>0.85	0.92	0.68<br>0.96	0.93	0.42<br>0.76	0.95

Abbreviations: NMQ-E, Nordic Musculoskeletal Questionnaire (Extended version); $ICC_{(2,1)}$, intraclass correlation coefficient (two-way random model, single measures, absolute agreement); 95%CI, 95% confidence interval; SEM, standard error of measurement, κ, kappa coefficient; κ/κ_{max}, the proportion of maximum kappa achieved; P_o, proportion of observed agreement.

problems with κ, concerns have been raised about assigning descriptive subdivisions[12] that are essentially arbitrary[6] and take into account only κ values rather than a range of indices. In consideration of all reliability statistics presented here, we believe the NMQ-E provides sufficiently reliable data for use as a screening instrument to quantify the prevalence and repercussions of musculoskeletal pain. There were instances where individual data items demonstrated poor repeatability, but these were uncommon and overall all questions exhibited acceptable stability of responses.

The NMQ-E produces sufficiently consistent responses when completed via self-administration and interview, evidenced by mean κ/κ_{max} ranging from .76 to 1.00 for dichotomous questions and mean $ICC_{(2,1)}$ = 0.90 (95% CI .57–1.00) for age of onset of symptoms. This indicates that the less expensive method of self-completion provides similar responses to those gained by physiotherapists during personal interviews, which has important financial implications for investigators who are planning a research study. In our study, validating NMQ-E responses with data obtained during physiotherapist interview found 0 to 24% divergent responses. This is similar to the finding of Kuorinka et al[25] who found 0 to 20% response discrepancy when validating NMQ responses with clinical history in small cohorts of medical secretaries (n = 19) and railway maintenance workers (n = 20).

The data for observed proportion of positive and negative agreement in this study suggest that the stability of negative responses was much greater than for positive answers. However, the divergent proportions may be a consequence of low prevalence. Low P_{pos} usually occurred where there was very low (or 0%) frequency of positive responses. Cicchetti and Feinstein[10] note that when the frequency of consistent positive and negative responses are dissimilar in the fourfold concordance table, then inequality is expected for P_{pos} and P_{neg} unless agreement is perfect.

The instability of κ in the presence of low prevalence is evident in our data. As outlined in Table 6, annual prevalence of sick leave is lower than annual prevalence of trouble in the low back region. Despite greater consistency of responses for sick leave, the corresponding κ value is much less than for annual prevalence of trouble. This result occurs because where prevalence is low, the proportion of agreement expected by chance (P_e) is high, and the chance-correction process performed in the calculation of κ[19,30] converts a high P_o into a low κ.[10] Therefore, a population with greater prevalence of the phenomena under study could yield higher κ statistics than a population with lower prevalence despite the same number of divergent responses. The κ/κ_{max} statistic corrects for the prevalence issue, as evidenced by the κ for annual prevalence of trouble data equating to only 77% of κ_{max} although data for annual sick leave attained κ_{max}. These data highlight the importance of examining test–retest reliability using a range of indices, particularly when the phenomena under study are either highly prevalent or rare.

Table 5. **Reliability Coefficients for Self and Interview Administration of the NMQ-E in an Occupational Cohort (n = 31): Questions Regarding Consequences of Pain**

	Lifetime Hospitalization		Lifetime Changed Jobs or Duties		Annual Prevention of Normal Work		Annual Visit to Health Professional		Annual Medication		Annual Sick Leave	
	κ / κ/κ_{max}	P_o	κ / κ/κ_{max}	P_o	κ / κ/κ_{max}	P_o	κ / κ/κ_{max}	P_o	κ / κ/κ_{max}	P_o	κ / κ/κ_{max}	P_o
Neck	*	1.00	0.47	0.93	0.52	0.90	0.79	0.93	0.27	0.87	0.65	0.97
	*		1.00		0.62		1.00		0.42		1.00	
Shoulders	1.00	0.93	0.65	0.97	1.00	1.00	0.84	1.00	1.00	1.00	*	1.00
	1.00		1.00		1.00		1.00		1.00		*	
Upper back	0.00	0.97	0.00	0.97	0.65	0.97	0.63	0.93	*	1.00	*	1.00
	0/0		0/0		1.00		1.00		*		*	
Elbows	1.00	1.00	*	1.00	*	1.00	*	1.00	*	1.00	*	1.00
	1.00		*		*		*		*		*	
Wrists/hands	0.00	0.97	0.61	0.90	0.63	0.93	0.78	0.97	*	1.00	*	1.00
	0/0		0.70		1.00		1.00		*		*	
Low Back	*	1.00	0.84	0.97	0.10	0.76	0.85	0.93	0.71	0.93	0.00	0.93
	*		1.00		0.16		0.85		0.71		0/0	
Hips/thighs	1.00	1.00	0.63	0.93	*	1.00	0.78	0.97	0.65	0.97	0.00	0.97
	1.00		0.63		*		1.00		1.00		0/0	
Knees	1.00	1.00	0.63	0.97	1.00	1.00	0.78	0.97	*	1.00	0.00	0.97
	1.00		0.63		1.00		1.00		*		0/0	
Ankles/feet	0.71	0.93	0.42	0.86	0.00	0.97	0.84	0.97	0.00	0.97	*	1.00
	0.71		0.42		0/0		1.00		0/0		*	
Mean	0.67	0.98	0.53	0.94	0.41	0.95	0.79	0.96	0.33	0.97	0.16	0.98
	0.96		0.80		0.83		0.98		0.83		1.00	

Abbreviations: NMQ-E, Nordic Musculoskeletal Questionnaire (Extended version); κ, kappa coefficient; κ/κ_{max}, the proportion of maximum kappa achieved; P_o, proportion of observed agreement.

The observed proportion of agreement for the NMQ-E is greater than that identified for the NMQ. We found P_o = .83–1.00 in comparison to P_o = .77–1.00 found by Kuorinka et al[25] using preliminary versions of the original NMQ in 3 small (n $\leq$ 29) studies of workers. Dickinson et al[16] found P_o = .74–1.00 for an English-language NMQ when the survey was completed at a week interval by supermarket workers (n = 44). Although these studies provide some indication as to the reliability of the NMQ in occupational cohorts, interpretation of their data is limited as proportion of agreement is a simplistic statistic that is not corrected for chance.

Chance-corrected reliability statistics for the NMQ questions have been reported in 3 studies. The reliability of the Greek NMQ was examined with 50 primary health care patients at a 2-week interval, with all questions scoring $\kappa \geq .82$ except the 7-day prevalence of neck and elbow trouble (κ = .64).[5] The Brazilian Portuguese NMQ was self-administered by a mixed cohort (nursing students, patients, administration staff and academic staff) at a single-day interval, and provided κ coefficients of .48–1.0, with 88% of questions scoring $\kappa > 0.75$.[13] A study by Rosecrance et al[34] included modified NMQ questions in a composite ergonomic research questionnaire and examined reliability in 99 factory workers at an interval of 14 to 33 days. Questions had a different focus to those in the NMQ and NMQ-E because they inquired about only work-related symptoms and activity prevention. In that study, annual prevalence of job-related musculoskeletal symptoms had κ that ranged from .13 to .71. The limitation of these studies is the reporting of kappa in isolation, an unstable statistic which can be inflated or lowered due to data characteristics (see Statistical Analysis section).[19]

Table 6. **Data Illustrating the Influence of Prevalence and Expected Proportion of Agreement on the Kappa Statistic**

Data	P_o	P_e	Prevalence (%)*	κ(SE)	κ/κ_{max}	Divergent Responses (n)
Annual prevalence of trouble (low back)	0.84	0.51	56.4	0.69 (0.10)	0.77	9
Annual prevalence of sick leave (low back)	0.95	0.95	2.6	0.00 (0.00)	0/0	3

*Mean of 2 testing occasions.

There are a number of potential sources of discrepancy in findings. Because the Greek[5] and Brazilian[13] studies comprised patient samples, higher frequencies of positive responses in those cohorts as compared with the occupational samples in this study and that of Rosecrance et al[34] may have contributed to higher kappa. The format and complexity of the survey instrument may play a role, as Greek[5] and Brazilian[13] versions had fewer questions than the NMQ-E and the composite ergonomic research questionnaire.[34] The use of different definitions of trouble could affect the stability of responses, with de Barros and Alexandre[13] adding numbness to the definition used in the NMQ-E and other studies.[5,34] Since kappa statistics are vulnerable to change dependent upon prevalence and distribution of data, it is not recommended to directly compare κ between studies or populations[12,41] and it is not known whether varying coefficients represent a true difference in reliability or an artifact of data distribution.

The original NMQ has been utilized predominantly in cross-sectional descriptive studies,[8,29,37] but also longitudinal studies[23,31] and intervention studies.[2,21] Cohorts studied include occupational[7,9,17] and general populations[20,32] and also patient samples.[28] The NMQ-E has similar applications, and is able to collect richer data on the prevalence of musculoskeletal pain and its consequences. The NMQ-E can also be used to classify musculoskeletal pain severity. Serious back pain has been defined as pain which requires treatment or sick leave, whereas non-serious back pain occurs in the absence of these repercussions.[1] The NMQ-E can facilitate such classification of pain for use in longitudinal studies of disease outcome.

A possible limitation of this study was a short time interval between administrations of the NMQ-E. Short durations between test and retest potentially introduce a memory effect whereby recall of previous responses can inflate reliability coefficients. We accepted a short test–retest interval to minimize the opportunity for changes in symptom status, and because we believed a memory effect would be unlikely due to the volume of instruments completed by participants (including the NMQ-E, a demographic questionnaire, WL-26[3] and Oswestry Disability Index[33]). Our findings are congruent with those from previous investigations of the NMQ,[5,13,25,34] thus there is no evidence of a memory effect inflating our findings. In the Brazilian reliability study, de Barros and colleagues[13] similarly used a single-day interval. It would be useful to examine the reliability of the NMQ-E after a longer duration (such as 7 and 14 days) to determine whether reliability estimates are systematically reduced as the intervening interval is increased.

In this study, NMQ-E data was validated against self-reported information provided to physiotherapists during personal interview. Responses were not externally validated with medical records (of hospitalization, for example) or workplace records of sick leave and restricted duties. It was not possible to examine concurrent validity via the standard approach since the NMQ-E does not measure a single construct and has no overall score with which to correlate with other established instruments. Examination of internal consistency was not appropriate since each NMQ-E question is unique and is not necessarily expected to be related to other questions. Future research should test the validity of NMQ-E responses against medical and workplace records (selecting a sample where such information is accessible) and could also investigate content validity to ensure that all important features of musculoskeletal trouble are incorporated in the tool. The reproducibility of online administration methods and the reliability of the NMQ-E in another occupational or general population should also be determined.

In summary, this study presents an extended English language version of the Nordic Musculoskeletal Questionnaire. The NMQ-E produces reliable data regarding the onset, prevalence and consequences of musculoskeletal pain in an educated occupational cohort and can be administered by self-completion and personal interview. The NMQ-E can be applied as a screening instrument for musculoskeletal pain and related events in studies of occupational and general populations. This is the first study of the NMQ to present a comprehensive range of coefficients that substantiate the reliability of this well-utilized tool.

References

1. Adams M, Mannion A, Dolan P: Personal risk factors for first-time low back pain. Spine 24:2497-2505, 1999

2. Alexandre N, de-Moraes M, Correa-Filho H, Jorge S: Evaluation of a program to reduce back pain in nursing personnel. Rev Saude Pub 35:356-361, 2001

3. Amick Br, Lerner D, Rogers W, Rooney T, Katz J: A review of health-related work outcome measures and their uses, and recommended measures. Spine 25:3152-3160, 2000

4. Andersson K, Karlehagen S, Jonsson B: The importance of variations in questionnaire administration. Appl Ergon 18: 229-232, 1987

5. Antonopoulou M, Ekdahl C, Sgantzos M, Antonakis N, Lionis C: Translation and standardisation into Greek of the standardised general Nordic questionnaire for the musculoskeletal symptoms. Eur J Gen Pract 10:33-34, 2004

6. Armitage P, Berry G, Matthews J: Statistical Methods in Medical Research, Malden. Massauchusetts, Blackwell Science Ltd, 2002, pp 278-282

7. Bao S, Winkel J, Shahnavaz H: Prevalence of musculoskeletal disorders at workplaces in the People's Republic of China. Int J Occup Saf Ergon 6:557-574, 2000

8. Choobineh A, Rajaeefard A, Neghab M: Association between perceived demands and musculoskeletal disorders among hospital nurses of Shiraz University of Medical Sciences: a questionnaire survey. Int J Occup Saf Ergon 12: 409-416, 2006

9. Choobineh A, Tabatabaei S, Mokhtarzadeh A, Salehi M: Musculoskeletal problems among workers of an Iranian rubber factory. J Occup Health 49:418-423, 2007

10. Cicchetti D, Feinstein A: High agreement but low kappa: II. Resolving the paradoxes. J Clin Epidemiol 43:551-558, 1990

11. Cohen J: A coefficient of agreement for nominal scales. Educ Psych Meas 20:37-46, 1960

12. Cook R: Kappa and its dependence on marginal rates, in Armitage P and Colton T (eds): The Encyclopedia of Biostatistics. New York, NY, Wiley, 1998, pp 2166-2168

13. de Barros E, Alexandre N: Cross-cultural adaptation of the Nordic musculoskeletal questionnaire. Int Nurs Rev 50: 101-108, 2003

14. de Vet H, Terwee C, Knol D, Bouter L: When to use agreement versus reliability measures. J Clin Epidemiol 59: 1033-1039, 2006

15. Deakin J, Stevenson J, Vail G, Nelson J: The use of the Nordic questionnaire in an industrial setting: A case study. Appl Ergon 25:182-185, 1994

16. Dickinson C, Campion K, Foster A, Newman S, O'Rourke A, Thomas P: Questionnaire development: An examination of the Nordic Musculoskeletal questionnaire. Appl Ergon 23:197-201, 1992

17. Dovrat E, Katz-Leurer M: Cold exposure and low back pain in store workers in Israel. Am J Ind Med 50:626-631, 2007

18. Dunn G: Design and analysis of reliability studies: the statistical evaluation of measurement errors. London, UK, Edward Arnold, 1989, pp 30-38

19. Feinstein A, Cicchetti D: High agreement but low kappa: I. The problems of two paradoxes. J Clin Epidemiol 43: 543-549, 1990

20. Hagen K, Svebak S, Zwart J: Incidence of musculoskeletal complaints in a large adult Norwegian county population. The HUNT Study. Spine 31:2146-2150, 2006

21. Hartvigsen J, Lauritzen S, Lings S, Lauritzen T: Intensive education combined with low tech ergonomic intervention does not prevent low back pain in nurses. Occup Environ Med 62:13-17, 2005

22. Hofmann F, Stossel U, Michaelis M, Nubling M, Siegel A: Low back pain and lumbago-sciatica in nurses and a reference group of clerks: Results of a comparative prevalence study in Germany. Int Arch Occup Environ Health 75: 484-490, 2002

23. Josephson M, Lagerstrom M, Hagberg M, Wigaeus Hjelm E: Musculoskeletal symptoms and job strain among nursing personnel: A study over a three year period. Occup Environ Med 54:681-685, 1997

24. Knibbe JJ, Friele RD: Prevalence of back pain and characteristics of the physical workload of community nurses. Ergonomics 39:186-198, 1996

25. Kuorinka I, Jonsson B, Kilbom A, Vinterberg H, Biering-Sorensen F, Andersson G, Jorgensen K: Standardised Nordic questionnaires for the analysis of musculoskeletal symptoms. Appl Ergon 18:233-237, 1987

26. Lagerstrom M, Wenemark M, Hagberg M, Wigaeus Hjelm E: Occupational and individual factors related to musculoskeletal symptoms in five body regions among Swedish nursing personnel. Int Arch Occup Environ Health 68:27-35, 1995

27. Landis J, Koch G: The measurement of observer agreement for categorical data. Biometrics 33:159-174, 1977

28. Larsson U: Influence of weight loss on pain, perceived disability and observed functional limitations in obese women. Int J Obes 28:269-277, 2004

29. Lee H, Wilbur J, Conrad K, Mokadam D: Work-related musculoskeletal symptoms reported by female flight attendants on long-haul flights. Aviat Space Environ Med 77: 1283-1287, 2006

30. Maclure M, Willett W: Misinterpretation and misuse of the kappa statistic. Am J Epidemiol 126:161-169, 1987

31. Maul I, Laubli T, Klipstein A, Krueger H: Course of low back pain among nurses: A longitudinal study across eight years. Occup Environ Med 60:497-503, 2003

32. Murphy S, Buckle P, Stubbs D: A cross-sectional study of self-reported back and neck pain among English schoolchildren and associated physical and psychological risk factors. Appl Ergon 38:797-804, 2007

33. Roland M, Fairbank J: The Roland-Morris Disability Questionnaire and the Oswestry Disability Questionnaire. Spine 25:3115-3124, 2000

34. Rosecrance J, Ketchen K, Merlino L, Anton D, Cook T: Test-retest reliability of a self-administered musculoskeletal symptoms and job factors questionnaire used in ergonomics research. Appl Occup Environ Hyg 17:613-6121, 2002

35. Shrout P, Fleiss J: Intraclass correlations: Uses in assessing rater reliability. Psychol Bull 86:420-427, 1979

36. Sim J, Wright C: The kappa statistic in reliability studies: use, interpretation, and sample size requirements. Phys Ther 85:257-268, 2005

37. Smith D, Mihashi M, Adachi Y, Koga H, Ishitake T: A detailed analysis of musculoskeletal disorder risk factors among Japanese nurses. J Safety Res 37:195-200, 2006

38. Streiner D, Norman G: Health measurement scales: A practical guide to their development and use. Oxford, Oxford University Press, 2003, pp 141-143

39. Traub R, Rowley G: Understanding reliability. Educ Meas Issues Prac 10:37-45, 1991

40. Trinkoff AM, Lipscomb J, Geiger-Brown J, Brady B: Musculoskeletal problems of the neck, shoulder, and back and functional consequences in nurses. Am J Ind Med 41: 170-178, 2002

41. Viera A, Garrett J: Understanding interobserver agreement: The kappa statistic. Fam Med 37:360-363, 2005

42. Walker B, Muller R, Grant W: Low back pain in Australian adults. Prevalence and associated disability. J Manipulative Physiol Ther 27:238-244, 2004

43. Weir J: Quantifying test-retest reliability using the intraclass correlation coefficient and the SEM. J Strength Cond Res 19:231-2340, 2005

Extended Nordic Musculoskeletal Questionnaire (NMQ-E)

How to answer the questionnaire:

Please answer by putting a cross in the appropriate box - one cross for each question. Answer every question, even if you have never had trouble in any part of your body. Please answer questions from left to right before going down to the next body region. This picture shows how the body has been divided. Limits are not sharply defined and certain parts overlap. You should decide for yourself which part (if any) is or has been affected.

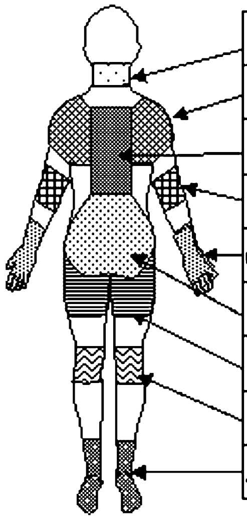

	Have you **ever** had trouble (ache, pain or discomfort) in:	**If 'No', go on to the next body region. If 'Yes', please continue**	At the time of initial onset of the trouble, what was your age?	Have you **ever** been hospitalised because of the trouble?	Have you **ever** had to change jobs or duties (even temporarily) because of the trouble?	Have you had trouble (ache, pain, discomfort) at any time during the **last 12 months?**	**If 'No', go on to the next body region. If 'Yes', please continue**	Have you had trouble (ache, pain, discomfort) at any time during the **last month (4 weeks)?**	Have you had trouble (ache, pain, discomfort) **today?**	During the **last 12 months** have you at any time: been prevented from doing your normal work (at home or away from home) because of the trouble?	seen a doctor, physio-therapist, chiropractor or other such person because of the trouble?	taken medication because of the trouble?	taken sick leave from work/studies because of the trouble?
NECK	☐ No ☐ Yes		___ years	☐ No ☐ Yes	☐ No ☐ Yes	☐ No ☐ Yes		☐ No ☐ Yes	☐ No ☐ Yes	☐ No ☐ Yes	☐ No ☐ Yes	☐ No ☐ Yes	☐ No ☐ Yes
SHOULDERS	☐ No ☐ Yes		___ years	☐ No ☐ Yes	☐ No ☐ Yes	☐ No ☐ Yes		☐ No ☐ Yes	☐ No ☐ Yes	☐ No ☐ Yes	☐ No ☐ Yes	☐ No ☐ Yes	☐ No ☐ Yes
UPPER BACK	☐ No ☐ Yes		___ years	☐ No ☐ Yes	☐ No ☐ Yes	☐ No ☐ Yes		☐ No ☐ Yes	☐ No ☐ Yes	☐ No ☐ Yes	☐ No ☐ Yes	☐ No ☐ Yes	☐ No ☐ Yes
ELBOWS	☐ No ☐ Yes		___ years	☐ No ☐ Yes	☐ No ☐ Yes	☐ No ☐ Yes		☐ No ☐ Yes	☐ No ☐ Yes	☐ No ☐ Yes	☐ No ☐ Yes	☐ No ☐ Yes	☐ No ☐ Yes
WRISTS/HANDS	☐ No ☐ Yes		___ years	☐ No ☐ Yes	☐ No ☐ Yes	☐ No ☐ Yes		☐ No ☐ Yes	☐ No ☐ Yes	☐ No ☐ Yes	☐ No ☐ Yes	☐ No ☐ Yes	☐ No ☐ Yes
LOW BACK	☐ No ☐ Yes		___ years	☐ No ☐ Yes	☐ No ☐ Yes	☐ No ☐ Yes		☐ No ☐ Yes	☐ No ☐ Yes	☐ No ☐ Yes	☐ No ☐ Yes	☐ No ☐ Yes	☐ No ☐ Yes
HIPS/THIGHS	☐ No ☐ Yes		___ years	☐ No ☐ Yes	☐ No ☐ Yes	☐ No ☐ Yes		☐ No ☐ Yes	☐ No ☐ Yes	☐ No ☐ Yes	☐ No ☐ Yes	☐ No ☐ Yes	☐ No ☐ Yes
KNEES	☐ No ☐ Yes		___ years	☐ No ☐ Yes	☐ No ☐ Yes	☐ No ☐ Yes		☐ No ☐ Yes	☐ No ☐ Yes	☐ No ☐ Yes	☐ No ☐ Yes	☐ No ☐ Yes	☐ No ☐ Yes
ANKLES/FEET	☐ No ☐ Yes		___ years	☐ No ☐ Yes	☐ No ☐ Yes	☐ No ☐ Yes		☐ No ☐ Yes	☐ No ☐ Yes	☐ No ☐ Yes	☐ No ☐ Yes	☐ No ☐ Yes	☐ No ☐ Yes

Appendix 1. The Extended Nordic Musculoskeletal Questionnaire (NMQ-E).
