# Supplementary material for: Prevalence of work related musculoskeletal disorders (WMSDs) and ergonomic risk assessment among readymade garment workers of Bangladesh: A cross sectional study
Source: PLoS One. 2018 Jul 6;13(7):e0200122. doi: 10.1371/journal.pone.0200122 (PMC6034848; doi:10.1371/journal.pone.0200122)
Supplement: S1 File — (PDF) [file pone.0200122.s002.pdf]

BRAC UNIVERSITY

JPG SCHOOL OF PUBLIC HEALTH
JAMES P. GRANT

KNOWLEDGE AND KNOW-HOW FOR HEALTH EQUITY

Date: 18 November, 2015

**Ethics Reference No:**<br>*Please quote this ref on all correspondence*	**69**
**Project Title:**	**Assessing long term health effects of working conditions among Ready Made Garment (RMG) workers in urban Bangladesh**
**Principal Investigator:**	**Dr. Malabika Sarker**

Thank you for submitting your application which was considered by the James P Grant School of Public Health, BRAC University Ethical Review Committee (ERC). The following documents were reviewed:

1. Ethical Review Checklist
2. Research Proposal
3. Consent Form
4. Questionnaires

The Ethical Review Committee approves this study from an ethical point of view upon the addressing by the researchers of the concerns as raised by the ERC affiliates.

Approval is given for three years. Projects, which have not commenced within two years of original approval, must be re-submitted to ERC. You must inform ERC when the research has been completed.

Any serious adverse events or significant change which occurs in connection with this study and/or which may alter its ethical considerations must be reported immediately to the ERC.

Approval is given on the understanding that the 'Guidelines for Ethical Review' are adhered to.

Yours sincerely,
Professor Syed Masud Ahmed
Member of ERC
James P Grant School of Public Health,
BRAC University

68, Shahid Tajuddin Ahmed Sharani, Level-6, icddr,b Building, Mohakhali, Dhaka-1212, Bangladesh
Phone:880-2-9827501-4 Fax:880-2-8810383 http://sph.bracu.ac.bd; www.jpgsph.org
